# Ivermectin in veterinary medicine: a narrative review of antiparasitic efficacy, resistance evolution, antiviral evidence, and One Health implications

**DOI:** 10.3389/fvets.2026.1833022

**Published:** 2026-06-03

**Authors:** Matthew T. J. Halma, Joseph Varon, Valeria Garcia, Sidra Hassaan, Jack Tuszynski

**Affiliations:** 1 Open Source Medicine OÜ, Tallinn, Estonia; 2 Independent Medical Alliance, Washington, DC, United States; 3 Universidad Autónoma de Baja California, Mexicali, Mexico; 4 Department of Physics, University of Alberta, Edmonton, AB, Canada; 5 Politecnico di Torino, Torino, Italy

**Keywords:** anthelmintic resistance, ecotoxicology, Ivermectin, macrocyclic lactones, One Health, veterinary medicine

## Abstract

Ivermectin is a common macrocyclic lactone that is proven to be active against a wide range of parasites in veterinary medicine. This article presents a narrative review that combines evidence on the antiparasitic effects of ivermectin, its therapeutic use in animals, the development of resistance, preliminary antiviral studies, and ecological consequences within a One Health framework. A comprehensive literature search was conducted through PubMed, Scopus, Web of Science, and Google Scholar for literature published between 1980 and 2025, and the evidence was synthesized qualitatively. Its main antiparasitic effect is mediated via glutamate-gated chloride channels in invertebrates, resulting in neuromuscular paralysis. Clinical benefit of established antiparasitic indications is best evidenced, but the results of treatment depend on the species of the parasite, the host species, the formulation, dosing conditions, and the baseline resistance condition. The antiviral effects reported are mostly exploratory, with most being the results of *in vitro* or preclinical studies, and are not a proven clinical activity in veterinary practice. Evidence on the environment also suggests that ivermectin residue may cause negative effects on non-target invertebrates, and thus stewardship-based dosing, resistance surveillance, and reducing ecological exposure should be considered in a One Health system.

## 1 Introduction

Ivermectin has been utilized in veterinary medicine since the beginning of the 1980s and is still one of the most prevalent antiparasitic agents used against livestock and companion animals (1). Its known veterinary applications are in the control of gastrointestinal nematodes and several ectoparasites, but the effects of therapy vary by host and parasite species, formulation, dose regimen, and geographic resistance patterns (2). This is why it is better to consider ivermectin in species-specific and management-specific terms, instead of a set of equally effective interventions in all veterinary systems (1, 3, 4).

Its major antiparasitic action is binding to glutamate-gated chloride channels in invertebrates, elevating the chloride inflow, hyperpolarizing the membranes, and causing neuromuscular paralysis (2–4). Its therapeutic selectivity is aided by the relative lack of these channels in mammals, but safety and performance are still dose and host-dependent, as well as dependent on pharmacologic context (2, 4).

Ivermectin is a derivative of avermectins produced by *Streptomyces avermitilis* and was a significant breakthrough in the control of broad-spectrum parasites and a significant part of contemporary veterinary parasitology (2, 4). Meanwhile, intensive and extensive animal production systems have augmented emphasis on resistance selection and environmental exposure due to long-term and repeated utilization (1, 4, 5).

### 1.1 Significance of the study

In addition to its known antiparasitic indications, experimental actions under controlled laboratory or preclinical conditions are gaining growing scientific attention for ivermectin, such as antiviral, immunomodulatory, and anti-inflammatory effects. These are mechanistically separate effects of its antiparasitic effect and are typically caused by the modulation of host cellular pathways, instead of pathogen lethality (2). Notably, these observations are seen mostly *in vitro* or small-scale animal models and cannot be considered to be similar to the established antiparasitic effect of ivermectin.

Moreover, within the macrocyclic lactone group, ivermectin has similar mechanistic properties to doramectin, eprinomectin, and moxidectin but the different agents differ in persistence, lipophilicity, tissue distribution, and residue properties (2–4). These compounds are, however, different in their pharmacokinetic behavior, lipophilicity, tissue persistence, and residue profiles. Moxidectin has a longer persistence than ivermectin and has been reported to be active in some nematode populations with reduced susceptibility as well, and eprinomectin is unique in the macrocyclic lactone group because it is used in dairy cattle with a minimum of milk residue issues (4). Doramectin offers a longer time of action in some livestock systems, whereas it has the same resistance-selection pressures as ivermectin (4). This wider macrocyclic lactone context is imperative in developing class-level interpretation of efficacy, resistance development, and ecological impact of ivermectin, which makes this review worthy at this point in time.

### 1.2 Scope

This narrative review concentrates on the proven veterinary antiparasitic use of ivermectin and clearly differentiates that evidence from antiviral and other non-antiparasitic observations. It also reflects on the effects of pharmacologic variability, development of resistance, and environmental impact on the practical application of ivermectin in animal health systems.

Due to the evidence base being *in vitro* studies, experimental animal models, field research, and applied veterinary reports, the review takes qualitative interpretation as opposed to a formal quantitative grading. Within this paradigm, *in vitro* research is considered mechanistic, and hypothesis-generating, experimental *in vivo* research is translational, and field or applied veterinary research is considered the most suitable for clinically actionable findings. The objective is to offer a balanced synthesis of veterinary evidence that separates the existing clinical evidence from preliminary or context-limited evidence (2, 4, 5).

### 1.3 Knowledge gap and aim

Although ivermectin has been used for over 40 years in veterinary medicine, the available literature is still disjointed. Published research is mostly concerned with single species, single parasite species, or single pharmacological actions. Consequently, mechanistic insights, clinical applications, resistance development, and environmental consequences are less integrated. Moreover, ivermectin’s mechanism of action is usually presented alone in the existing literature without any adequate connection to comparative pharmacokinetics, species-specific dosing, or ecological impact. There are also serious gaps in the standard reporting of the incorporation of resistance traits into the stewardship measures and the ecological impact of ivermectin residues in dung and soil food webs. Specifically, resistance in food-borne nematodes is steadily increasing, but mechanistic resistance information is hardly addressed in combination with applied dosing or sustainability models. In this connection, it should be emphasized that the novelty of this review is not in the identification of new effects, but in the synthesis of the existing evidence in traditionally discrete domains. This review aims to present molecular mechanisms, therapeutic uses in veterinary practice, resistance patterns, and environmental effects in a unified ‘One Health’ framework.

The thematic objectives of this review are:

1. Review the known antiparasitic processes and pharmacological characteristics of ivermectin in veterinary hosts.
2. Differentiate between validated clinical applications and experimental or preclinical findings.
3. Assess mechanisms of resistance and stewardship challenges in livestock and companion animals.
4. Determine ecological and environmental consequences of using ivermectin in the animal health system.

## 2 Methods

A structured literature search and qualitative synthesis were used to conduct the narrative review. The aim was to determine and analyze the useful veterinary literature in the mechanistic, therapeutic, resistance-related, pharmacologic, and ecological fields of ivermectin use and not to conduct a formal systematic review or meta-analysis. Based on this, the protocol registration and PRISMA-based reporting processes, duplicate independent screening, official inter-reviewer agreement evaluation, and study-level risk-of-bias instruments were not implemented. Rather, the review adopted qualitative interpretive methods to differentiate between mechanistic, preclinical, translational, and practice-relevant veterinary evidence.

PubMed, Scopus, Web of Science, and Google Scholar were searched to find the literature published between 1980 and 2025. Key search terms were ivermectin, veterinary, antiparasitic, antiviral, livestock, companion animals, ruminants, poultry, equine, resistance, macrocyclic lactones, pharmacokinetics, toxicity, ecotoxicology, and One Health. In PubMed, a representative Boolean search structure was constructed based on controlled vocabulary and free-text terms and modified to suit the other databases. The last literature search was performed in April 2025. Moreover, reference lists of the relevant full-text articles were screened manually to find additional literature of interest to the review scope.

Narrative synthesis relied mostly on original research, such as *in vitro* research, *in vivo* animal research, controlled field research, observational research, and applied veterinary pharmacology research with veterinary-relevant species. Research was included in the eligibility criteria in case it provided mechanistic, therapeutic, ecological, pharmacokinetic, or resistance-related data in animal health systems. Evidentiary synthesis was not applied to human-only studies, non-veterinary pharmacology studies, and those reports which had no clear relevance to the scope of the review. Only the selected review articles, overview papers, and guidance-oriented sources were retained when required to provide historical background, conceptual framing, or contextual interpretation and were not considered equal to primary evidence as part of the qualitative synthesis.

Since the literature included was extremely heterogeneous in terms of study design, species, interventions, and outcomes, quantitative pooling was not deemed the appropriate choice. Synthesis of the literature was thus done in a narrative manner with special focus on differentiating the known clinical evidence on antiparasitism from exploratory mechanistic or preclinical evidence. The review covered a total of 118 sources within the thematic domains. As this study involved an analysis of published literature, ethical approval was not required.

## 3 General veterinary use of ivermectin

Ivermectin is a popular veterinary antiparasitic compound with practical utility in livestock, equine, companion-animal, and selected small-mammal systems. In this section, the focus is on general trends of veterinary application, such as the host range, formulation-specific issues, and the clinical context where efficacy, resistance selection, and environmental exposure overlap. Instead of giving a disease-by-disease synthesis, it is a general practice-oriented introduction which sets up the more indication-oriented discussion that follows in the next section.

Ivermectin serves as a highly important veterinary drug that is widely used for its effectiveness against a wide range of parasite disease conditions occurring in livestock, pet animals, and wild animals. Its role in the effective management of gastrointestinal nematodes that are found in ruminants is most prominent in clinical research. Some of the most notable species of nematodes are *Cooperia* spp., *Ostertagia ostertagi,* and *Haemonchus contortus*, resulting in large-scale production losses worldwide. Its application in veterinary medicine has been linked to the advancement of animal health parameters and a decrease in production losses associated with parasites in particular management settings (4). These effects depend on the context and differ depending on the species, parasite burden, dosing schedule, and herd management. Currently, different meta-analyses have found that attempting treatment with ivermectin in sufficient doses can result in rates of treatment as high as 90%, a clinically important finding. The levels of reported efficacy vary according to the target parasite species, formulation and route of administration, baseline resistance status, and the clinical or parasitological endpoint on which the response to treatment is determined. However, such treatment is continually being threatened by a rising level of resistance. This has been especially seen in Haemonchus sp. in countries of Africa, South America, and Asia (5). Moreover, ivermectin is highly effective against ectoparasites (*Sarcoptes scabiei* and *Psoroptes ovis*) and against nematodes. These species infect sheep and cattle with mange (5).

Ivermectin is a common treatment for both swine-borne Mange (caused by the mite *Sarcoptes scabiei* var. *suis*), and also for the treatment of the gutworms, *Ascaris suum.* These parasites affect their feed efficiency, impair immune responses, and cause the pigs to suffer secondary infections. The use of ivermectin in swine herds has been associated with better feed conversion ratios and reduced morbidity (6). Likewise, in equine practice, ivermectin has been the central point of parasite control for more than three decades. Its *in-vitro* activity is wide (covering strongyles, *Parascaris equorum*, and bot larvae), which historically was a prime contributor of significant morbidity and mortality in foals and yearlings. Current guidelines focus on strategic evidence-based dosing strategies to help reduce resistance in equine strongyles, as this problem has started to be observed in some parts of the world (7).

Ivermectin also plays an important role in companion animal medicine. It is widely used as prophylaxis in dog and cat heartworm disease (*Dirofilaria immitis*), which otherwise may cause severe cardiopulmonary pathology, and monthly prophylaxis with ivermectin-based preparations is still in use in most areas (8). Ivermectin should not be used solely to prevent heartworms in companion animals. The range of ecto- and endoparasitism disease life cycles encompassed by small-animal parasitology is more extensive, and ivermectin-based combination regimens have also been implemented in dogs to prevent heartworm infections alongside hookworm and ascarid infection treatment (8). Ivermectin is also widely used to treat rabbit, guinea pig, and rodent ectoparasites, such as fur and ear mites (9). The use of ivermectin in these species reveals the versatility of ivermectin in a broad spectrum of veterinary practices when administered at therapeutic doses. Meanwhile, the disease-based synthesis that is introduced later in this review is more detailed for livestock-associated indications, mostly due to the abundance of literature available to be put into the current narrative synthesis, more thoroughly developed in food-producing animal systems than in companion-animal parasitology.

However, there have been increasing concerns over the ecological impacts. Ivermectin residues found in the feces do not disappear from the environment, and they are toxic for dung beetles, earthworms, and other aquatic invertebrate populations, a key aspect for nutrient dynamics and soil health (1, 10). Moreover, the effects of dosing with ivermectin and performance in the clinical performance of veterinary species, as well as formulations, differ significantly. There are differences in bioavailability, persistence, and tissue distribution of injectable, oral, and pour-on preparations, which can both affect efficacy and resistance selection. Differences in metabolism and fat partitioning between the species also complicate the procedure of extrapolating doses, and there is a need to use a different dose based on the species and the formulation itself, instead of assuming the same dose across species (2, 4). Based on these results, it is important that integrated parasite management strategies combining ivermectin with other levels of intervention must be considered to ensure that efficacy is achieved whilst minimizing the risk to the environment.

## 4 Disease-specific antiparasitic applications of ivermectin

This section gives an indication-based synthesis of some of the parasitic diseases and ectoparasitic infestations where ivermectin has found application in veterinary medicine. The emphasis in this section is on practical antiparasitic indications instead of on general practice trends, and the examples are aimed at demonstrating diversity in host range, evidence strength, and clinical context instead of being a complete catalogue of ivermectin uses around the world.

### 4.1 Scope of antiparasitic indications covered in this review

This subsection presents the selected parasitic indications that were chosen to undergo narrative synthesis and is supposed to contextualize the spectrum of veterinary settings, which will be addressed further (11, 12). Examples below are not meant to be a comprehensive catalogue of all world applications of ivermectin, but to show variability in disease burden, host setting, and applied veterinary relevance in the literature (13, 14). Nematodes of the gastrointestinal tract are still regarded as one of the most significant parasitic targets in livestock systems, though the overall impact of parasitic disease and its practical implications can significantly depend on the host species, production system, and geography (15, 16). It is due to this that the section below does not concentrate on a global ranking of all ivermectin applications but just on a few representative indications (17, 18). Ivermectin has been tested in a variety of parasitic disease and infestation contexts, as summarized in Table 1, but with varying levels of supportive evidence.

**Table 1:** Selected parasitic disease and infestation contexts relevant to ivermectin use in veterinary medicine.

Target parasite/Disease context	Illustrative burden/Epidemiologic context	Evidence category	Principal mechanism
*Hypoderma bovis, Hypoderma lineatum*	Estimates vary by region. (16) 40% of British cattle. (16)	Animal Trial: 100% efficacy in treating bovine hypodermosis (17)	Unknown, possibly GluCI-mediated toxicity (15)
*Cheyletiella* spp.	4% of rabbits in Dutch pet shops (23)	Animal Trial: Effective in animal trials (24, 25)	Unknown, possibly GluCI-mediated toxicity (15)
*Babesia* and *Theileria parasites*	~30% of horses (26)	Preclinical: IVM inhibits *Babesia* parasites in growth inhibitory assays (22)	Unknown
*Culicoides* spp.	~2/3 of cattle in a survey in Maharashtra state, India (144), is not common in North America.	Animal Trials (Conflicted): Conflicting data in veterinary trials, some trials showing efficacy (81), others showing no efficacy (145, 146)	Unknown
*Haemonchus contortus*	60% prevalence among cattle in an Ethiopian survey (147)	Animal Trials (148, 149)	IVM binds to the GluCI receptor (78)
*Sarcoptes scabiei* var. *bovis.*	Survey in Pakistan: 2% of cattle (150) Ethiopia: 22% of cattle (49)	Animal Trial: Eliminated from IVM-treated cattle (151)	IVM binds to the GluCI receptor (78)
*Linognathus vituli* and *Damalinia bovis*	4% of cattle in the Icelandic sample (152)	Animal Trial: *L. vituli* eliminated, D. bovis reduced (151)	IVM binds to the GluCI receptor (78)
*Boophilus decoloratus, Amblyomma hebraeum, Rhipicephalus appendiculatus, Rhipicephalus microplus,* and *Hyalomma* spp.	2% of cattle in Great Britain (153)	Animal Trial: Numbers reduced with subcutaneous IVM injection (151) exposure to ivermectin systemically related to decreased survival and oviposition (4).	IVM binds to GluCI receptor (78) Acaricidal activity in line with the antiparasitic neurotoxic effect of ivermectin (4).

### 4.2 Bovine hypodermosis

Bovine hypodermosis, a parasitic infection by *Hypoderma bovis* and *Hypoderma lineatum*, primarily affects cattle and is reported in up to 40% of British cattle (13). Ivermectin has shown high efficiency in controlled field trials, and in certain circumstances, treatment success rates have been reported to be above 90%. These, however, are dependent on the timing of administering, formulation, and region-specific patterns of parasite susceptibility, and should not be perceived as being universally applicable (17, 19). Furthermore, other mechanisms have also been put forward, such as oxidative stress in parasites or host-mediated immunomodulatory mechanisms (2), but these are hypothesis-generating and not as well confirmed as GGCC-mediated paralysis.

The larvae travel through connective tissues and then fixate under the dorsal skin, producing a series of warbles which cause hide damage, weight loss, and diminished milk yield. Until the advent of ivermectin, control was found to be achieved only by the use of topical insecticides and manual removal, both of which were labor-intensive and less effective. The advent of macrocyclic lactones such as ivermectin revolutionized the management of bovine hypodermosis, greatly reducing larval burdens and preventing economic losses in cattle herds. This not only helps to maintain hide value, but also to avoid problems like myiasis and secondary bacterial infections. Recent surveillance studies continue to highlight the impact of ivermectin-based control programs on reducing hypodermosis prevalence (19). A recent field survey was conducted in Turkey in which an emergence of reduced sensitivity of *Hypoderma* populations to macrocyclic lactones was reported, which points to the need to be cautious about when treatment occurs and to continue resistance monitoring in these cases (20). Nevertheless, ivermectin is the most reliable systemic intervention in large-scale eradication and control programs against hypodermosis in both dairy and beef cattle.

The appearance of decreased sensitivity of *Hypoderma* populations from the stewardship point of view highlights the importance of a prudent use of ivermectin and optimizing the timing of ivermectin administration and ongoing monitoring of resistance to maintain long-term efficacy and avoid unsustainable selection pressure.

### 4.3 Fur mites and mange

Fur mites include infestations caused by *Cheyletiella* spp., affecting rabbits and small mammals. A Dutch study showed infection of 4% of rabbits in a pet shop, with a high transmission rate (13). Animal trials have confirmed the ability of ivermectin to eliminate these parasites (14, 15). Although the mechanism is not yet fully understood, it likely involves glutamate-mediated excitotoxicity (18).

*Sarcoptes scabiei, Psoroptes ovis,* and *Chorioptes bovis* cause Mange, which is a significant ectoparasitic disease affecting a variety of livestock. These mites invade either the epidermis (Sarcoptes) or the skin (Psoroptes, Chorioptes), causing severe pruritus, hyperkeratosis, epidermal scaling, and skin thickening. This may develop into poor body condition and emaciation of pigs in chronic infestations, especially where the irritation is persistent, and there is poor intake of feed. *Sarcoptes scabiei* var. *suis* is a significant parasitic problem of swine production, infection of which is characterized by a decrease in feed ratio, deterioration of growth rates, and an increased risk of secondary bacterial infections. Ivermectin continues to be the foundation of mange control due to its systemic distribution and its action against both burrowing and surface-dwelling mites among acaricidal agents that are available. It acts by binding glutamate-gated chloride channels in mite nerve and muscle cells, leading to paralysis and subsequent death. A high cure rate of over 95% with injectable or pour-on preparations has been reported with significant clinical response in pruritus and skin lesions being observed in the 2–3 weeks of treatment (21). Ivermectin is also successful in the treatment of *Psoroptes* and *Sarcoptes* infections in small ruminants, but in intensively managed flocks, frequent repeated treatment is frequently necessary to eradicate the infection because of the pressure of reinfection and the life-cycle nature of the mite. Though the cases of ivermectin resistance in sheep are not much documented, the incidences have been recorded after long and continuous use, highlighting the necessity of the strategic treatment regimen and rotation of parasites protections (5).

### 4.4 Piroplasmosis (babesiosis and Theileriosis)

*Babesia* and *Theileria* species cause piroplasmosis, which is not a worldwide infection but is localized to certain geographical areas. Localized disease burden has been reported as high as 30% localized prevalence rates of serological and field surveys in horses (16). Due to its mode of transmission, it is a significant concern in endemic regions where ticks are prevalent. Preclinical data suggest that ivermectin may have therapeutic potential. In growth assays, ivermectin has been shown to inhibit the growth of Babesia parasites; however, the actual mechanism of action is speculative, and is not regarded as a major antipiroplasm effect (17). The imidocarb and diminazene aceturate are the primary treatment options, but ivermectin is more appropriately considered in this context as an indirect or adjunctive therapy, which may decrease the tick burden and subsequently the transmission pressure instead of as a primary antipiroplasm therapy. This is especially pertinent to *Rhipicephalus microplus,* which is a significant cattle tick in endemic production systems where tick-related parasitism is a contributor to health and productivity costs on a bigger scale (11). It has also been reported to transmit *Babesia* species, causing equine piroplasmosis in horses (11). The recent research studies used in the current review indicate that there is a possibility that systemic ivermectin administration can decrease the survival and oviposition of *R. microplus*, which can lead to the epidemiologic pressure of the tick-borne protozoal transmission (4). Nevertheless, these observations are viewed as a demonstration of ectoparasite-control relevance in an integrated management system, rather than demonstrating that ivermectin is a treatment option of choice in babesiosis or theileriosis itself (4). Furthermore, ivermectin has shown a possible mild inhibitory effect on the protozoan growth *in vitro*, albeit with lesser potency compared to the traditional antiparasitic anti-piroplasm compounds (22). However, the evidence based on these observations is mainly grounded on *in vitro* or adjunctive studies and cannot be construed as evidence of clinical efficacy on its own against protozoal infection.

### 4.5 *Culicoides* infestation and vector control

The *Culicoides* biting midges are common in subtropical and tropical areas. The infestation is not common in America, but a survey conducted in Maharashtra, India, indicated that the infestation was witnessed in about two-thirds of cattle (23). Veterinary experiments assessing the effect of treatment against *Culicoides* infestation have shown mixed results: some studies show significant effects with poorly specified mechanisms, others do not show a significant effect (24–26). Ivermectin administered orally or subcutaneously to ruminants and equines is ingested by midges during blood feeding and causes high mortality rates and fecundity of females. Endemic research in the field demonstrates that with the use of ivermectin on cattle, there is a lower success rate of midge feeding and fewer egg-laying rates, leading to localized declines in the density of vectors over time (27). This offers a direct reduction in biting pressure of livestock and can lower the risk of transmitting arboviruses. Notably, the ivermectin left behind in the blood of the hosts is deadly to midges up to 10–14 days after administration, which lasts longer than the treatment (28).

Ivermectin has certain applications in the field of equine medicine, especially against African horse sickness, spread by *Culicoides imicola* among other species. The experimental trials indicate that ivermectin use in horses lowers the midge survivorship and vector competence, which supports its use as an adjunct to the integrated control strategies in the environment where the use of the vaccine is not enough (29). Equally, a combination of environmental control and vaccination with ivermectin treatment of cattle during outbreaks of bluetongue has been linked with a reduction in the circulation of viruses. Nonetheless, ivermectin is not fully effective in controlling the *Culicoides* populations; the effects are usually partial, and there are fears of environmental sustainability and other implications in non-target arthropods such as pollinators and dung-related beneficial species (1). Moreover, *Culicoides* species have been noted to vary in susceptibility (30). These restrictions show that the use of ivermectin is best suited in integrated management of vectors in combination with environmental management, repellents, and vaccines. In other modelling studies, it is hypothesized that synchronized mass treatment of livestock in dense farming systems can have more extensive, population-level impacts by lowering the density and risk of vectors, and the effects of these programs are context-specific and depend on coordinated efforts (28).

### 4.6 Other ectoparasitic infestations

Ivermectin has been used successfully against mites and ticks, and more generally as a shield from other ectoparasites impacting animal health and animal production. Similarly, lice (*Linognathus vituli* and *Damalinia bovis*) and horn fly (*Haematobia irritans*) infestations in cattle are successfully treated by ivermectin, resulting in greater weight gain and milk yields. The use of ivermectin in poultry is also limited by its status of regulatory approval, withdrawal period requirements, and considerations of residues, and should therefore be used cautiously and species-specifically. Off-label use of ivermectin in poultry has been carried out for mite control, such as *Dermanyssus gallinae*, but dosing and/or effluent residue concerns should be closely regulated (2). The broad interpretability of ivermectin to ectoparasitic infestation is an indication of its importance as an essential veterinary tool, but it also indicates the need for sustainable use to reduce ecological effects and maintain its efficacy for the longer term.

## 5 Veterinary viral diseases

The reviewed studies in this section represent a broad spectrum of veterinary causative agents of disease, mechanisms of action, and outcome measures. Despite several findings, the precise mechanism through which ivermectin interferes with most of the viral replication cycles remains unknown. Further research is needed to elucidate its mode of action, but the existing evidence is to be viewed as preliminary and not evidence of proven antiviral use in veterinary disease management. The combination of viral agents is aimed at describing experimental exploration coverage, and not to indicate comparable or clinically significant antiviral effectiveness. The majority of antiviral effects reported are of *in vitro* origin or of exploratory models, often at dosages unattainable using standard veterinary dosing, and *in vivo* and field results are scarce, inconsistent, or nonexistent. Based on this, antiviral results are viewed as mechanistic or hypothesis-generating data, and the known veterinary value of ivermectin has remained antiparasitic, and not antiviral. Table 2 outlines the antiviral effects, proposed mechanisms, and strength of evidence of ivermectin on various animal viruses.

**Table 2:** Exploratory antiviral studies of ivermectin in animal viruses: epidemiology, experimental context, experimental evidence category, and proposed mechanism.

Virus	Worldwide incidence	Experimental context	Experimental evidence category	Proposed mechanism
Foot and Mouth Disease Virus (FMDV)	Worldwide: 1.8% of cattle, 1.5% of goats, 1.4% of pigs, 1.0% of sheep (31)	*In vitro* + *in vivo*	Preclinical data (32). Lack of efficacy against serotype O in mice (33)	Unknown
Hendra Virus (HeV)	Humans: 1.68 cases per year 0.13 deaths per year, localized to Australia (1994–2015) (53) Horses: 3 cases per year, 2.7 deaths per year (53)	*In vitro*	Preclinical (54)	Inhibition of importin α (54)
Newcastle Disease Virus (NDV)	In an Indian study, Newcastle disease affected 14% of commercial birds (46) Not common in North America and Europe, controlled by vaccination (47)	*In vitro* + animal trial	Preclinical: Antiviral effect at 100 μg/mL IVM, but also cytotoxicity (48) Animal Trial: Laying hens with NDV showed improvement when treated with IVM (50)	Potentially importin α1/β1 (48)
Infectious Laryngotracheitis Virus (ILTV)	Prevalence of 13% of commercial poultry in Ethiopia (35)	Animal trial	Animal Trial: Laying hens with ILTV showed improvement when treated with IVM (34)	Unknown
Equine Herpesvirus (EHV)	EHV-1: >60% of horses worldwide (55) EHV-4: ~66% of horses (56)	*In vitro*	Preclinical: Some reduction of replication in EHV-1 strain Jan-E replication but not strain Rac-H (57)	Importin α/β disruption (57)
Pseudorabies Virus (PRV)	3% of domestic pigs in a Croatian study (34)	*In vitro*	Preclinical: Reduction of PRV proliferation in BHK-21 cells (35) and Vero cells (36)	Inhibition of DNA polymerase UL42 (35)
Porcine Circovirus 2 (PCV2)	57% PCR test positivity in Chinese study (37)	*In vitro* + *in vivo*	Preclinical: Inhibition of PCV2 replication in PK-15 cells (38) Animal Trial: Mitigation of PCV2 infection in piglets (38)	Disruption of nuclear import (38)
Bovine Respiratory Syncytial Virus (BRSV)	10.8% (58)	*In vitro*	Preclinical: Inhibition of BRSV replication *in vitro* (MDBK cell line) (59)	Postulated: Importin α/β inhibition
Bovine Parainfluenza Virus Type 3 (BPIV-3)	0.64% (60) 13.5% (58)	*In vitro*	Preclinical: Inhibition of BPIV-3 replication *in vitro* (MDBK cell line) (59)	Postulated: Importin α/β inhibition
Bovine Herpesvirus 1 (BoHV-1)	5.4% (62)	*In vitro*	Preclinical: Inhibition of BoHV-1 replication *in vitro* (MDBK cell line) (59)	Postulated: Importin α/β inhibition
Bovine Coronavirus (BCoV)	32.4% (58)	*In vitro*	Preclinical: Inhibition of BCoV replication *in vitro* (MDBK cell line) (59)	Postulated: Importin α/β inhibition
Bovine Viral Diarrhea Virus (BVDV)	≤0.8% in Europe, North America, and Australia to >1.6% in West Asia (61)	*In vitro*	Preclinical: Inhibition of BVDV replication *in vitro* (MDBK cell line) (59)	Postulated: Importin α/β inhibition
Varicellovirus Bovinealpha 1 (BoAHV-1)	21.43% (63)	*In vitro*	Preclinical: Inhibition of BVDV replication *in vitro* (MDBK and BT cell lines) (64)	Unknown
Malignant Catarrhal Fever (alcelaphine herpesvirus 1) (AHV-1)	In Kenya, causes loss of up to 10% of cattle herds per year (65)	*In vitro*	Preclinical: Inhibition of viral replication *in vitro* (BT1621 cell line) (66)	Potentially inhibition of nuclear transport (66)
Infectious Bovine Rhinotracheitis Virus (IBRV)	3.4% of cattle in a Mexican survey (67)	*In vivo*	Animal Trial: Inhibition of viral replication in a rabbit model (68)	Unknown, but IVM did not affect viral binding and entry (68)
Lumpy Skin Disease Virus (LSDV)	54% of cattle in Africa (39)	*In vitro*	Preclinical: Strong inhibition *in vitro* at the viral replication stage (99.8%), attachment stage (68.4%), and penetration stage (57.8%) (40)	Unknown
Sheep Pox (SPPV)	2% (Northern Nigeria) (41) 16% (Amhara, Ethiopia) (42)	*In vitro*	Preclinical: Strong inhibition *in vitro* at the viral replication stage (99.9%), weak inhibition at the attachment stage (25.1%), and no inhibition at the penetration stage (0.0%) (40)	Unknown
Porcine Epidemic Diarrhea Virus (PEDV)	Resulted in a loss of almost 10% of US domestic pig production (43)	*In vitro*	Preclinical: Inhibition of PEDV replication in Vero cells (36, 44) and porcine LLC-PK1 cells (36)	Potential disruption of nuclear import (36)
Swine Vesicular Stomatitis Virus (VSV)	Low incidence (0.2%) in feral swine in the USA, possibly endemic to other regions (45)	*In vitro*	Preclinical: Inhibition of VSV in Vero cells (36)	Unknown
Peste des Petits Ruminants Virus (PPRV)	79–100% of cattle (Sudan) (61)	*In vitro*	Preclinical: Inhibition of replication in Vero cells, but limited effect on viral entry (63)	Unknown
Viral Hemorrhagic Septicemia Virus (VHSV)	72.4% of cattle (65)	*In vitro* + *in vivo*	Preclinical: Inhibition in EPC cells (66) Animal Trial: Better survival rates in VSHV-infected olive flounder (66)	Unknown
Porcine Reproductive and Respiratory Syndrome Virus (PRRSV)	11 to 32% of pigs (66)	*In vitro* + *in vivo*	Preclinical: Inhibition of replication in PAM-pCD163 cells (67) Animal Trials: May have reduced lung lesions in veterinary trials (68)	Inhibition of viral entry, no inhibition of nuclear import (67)
Usutu Virus (USUV)	105 cases in humans, several outbreaks in birds and mosquitoes (69)	*In vitro*	Preclinical: Inhibition in Vero CCL-81, A549 and TME-R cells (70)	Inhibition of flaviviral non-structural protein 3 (71)
Parvoviruses (PV)	Porcine parvoviruses, 19.6% (72)	*In vivo*	Animal Trial: Lower levels of hypertrophied nuclei in parvovirus-infected crayfish administered IVM (73)	Putatively nuclear localization disruption (73)
Papillomaviruses (PV)	Papillomavirus affects 4.4% of cattle (74)	In vivo / observational	Animal Trial: No remission from bovine cutaneous papillomatosis in the control group, 83% remission in IVM groups (75) Cattle given IVM had regression of cutaneous papilloma (74) Improved healing of udder papillomaviruses in goats (75) Improvement in equine papillomatosis symptoms with oral IVM administration (76) Complete remission in heifer and calf papillomatosis (77)	Unknown
Avian Infectious Bronchitis Virus (IBV)	~40% (Spain) broiler chickens in (51)	*In vitro*	Preclinical (negative): Lack of efficacy in preclinical experiments (52)	N/A
Bluetongue Virus (BTV)	1% of cattle in a Florida sample (63)	*In vitro*	Preclinical: No effect observed *in vitro* (64)	N/A

### 5.1 Viral diseases of livestock

Foot and Mouth Disease Virus (FMDV), Porcine Circovirus 2 (PCV2), Porcine Reproductive and Respiratory Syndrome Virus (PRRSV), Pseudorabies Virus (PRV), Lumpy skin disease virus (LSDV), Sheep pox (SPPV), Porcine epidemic diarrhea virus (PEDV), Swine vesicular stomatitis virus (VSV), and Bluetongue Virus (BTV) are some of the most prominent viral diseases in swine and cattle production systems for which Ivermectin’s role has been studied for.

FMDV affects livestock worldwide, with a prevalence reported in 1.8% of cattle, 1.5% of goats, 1.4% of pigs, and 1.0% of sheep (31). Preclinical studies have tried exploring the potential beneficial effects of ivermectin as an antiviral agent, but its efficacy remains unproven, particularly with serotype O in mice (32, 33). A Croatian study found the PRV in 3% of domestic pigs (34). Reduced PRV replication in BHK- 21 and Vero cell lines following exposure to ivermectin has been reported *in vitro* (35, 36).

PCV2 is a significant concern in swine populations, with a Chinese study reporting a 57% positivity rate using PCR testing (37). Ivermectin has been shown to prevent PCV2 replication in PK-15 cells, although the existing evidence is preclinical and not proven as having clinical efficacy in swine (38). Its mechanism of action is believed to involve the disruption of nuclear import, which is essential for viral replication (38).

LSDV represents another significant veterinary concern in Africa, affecting approximately 54% of the cattle population (39). Recent *in vitro* experiments documented significant attenuation of LSDV under experimental conditions, such as replication, attachment, and penetration; nonetheless, these data are still preclinical and not to be construed as clinical antiviral effects (40).

SPPV is yet another important veterinary pathogen with a reported prevalence of 2% in Northern Nigeria and up to 16% in the Amhara region of Ethiopia, highlighting its regional impact on livestock health and productivity (41, 42). *In vitro* studies have shown that ivermectin exhibits a strong inhibitory effect on viral replication, reducing it by 99.9%. Experimental evidence from *in vitro* studies has shown significant inhibition of SPPV replication conditions, but a small effect on attachment and no inhibition on viral penetration (40).

PEDV is a highly contagious pathogen that primarily affects swine, leading to severe diarrheal disease, dehydration, and high mortality rates, particularly in neonatal piglets (43). *In vitro* experiments have indicated that PEDV replication is inhibited in Vero and porcine LLC-PK1 cell lines, although this is preclinical evidence that is not proven to provide clinical benefit in swine (36, 44). The proposed mechanism of action is believed to involve the disruption of nuclear import, a crucial process for viral replication and host cell manipulation (36).

VSV is a viral pathogen that primarily affects swine and other livestock, causing vesicular lesions that can be easily confused with those of foot-and-mouth disease. In the United States, the incidence of feral swine is relatively low, with a reported prevalence of only 0.2% (45). However, evidence suggests that VSV may be endemic in other regions, where its impact on animal health and agricultural productivity remains a concern. *In vitro* studies have recently demonstrated inhibition of VSV replication in Vero cells, but this is merely a primary mechanistic observation and not indicative of a veterinary antiviral effect (36).

Differences in depth of viral pathogens indicate differences in experimental evidence available and not clinical relevance. Reported prevalence data are provided to put disease burden in perspective, rather than antiviral activity, and common mechanistic motifs, typically an interference with host nuclear importation, are conceptualized. Since the antiviral effects have mostly been obtained in *in vitro* and exploratory preclinical laboratory research, usually at non-translatable concentrations, the results are considered to be mechanistic or hypothesis-generating and not indicative of clinical activity in livestock.

### 5.2 Viral diseases of poultry

Ivermectin has also been experimentally investigated as a potential treatment of several poultry viral pathogens, such as Newcastle Disease Virus (NDV), Infectious Laryngotracheitis Virus (ILTV), and Avian Infectious Bronchitis Virus (IBV), which are major causes of health problems in poultry production systems. Existing literature on the subject of ivermectin as an antiviral agent in avian species is narrow and uneven in its design and should be viewed with caution.

NDV is a significant avian pathogen in the world, but its prevalence is relatively low in North America and Europe, where vaccination is widely applied. Conversely, NDV prevalence as high as up to 14% was reported in a commercial poultry business in a study conducted in India (46, 47). *In vitro* experimental studies have shown that ivermectin, when used at concentrations of about 100 mg/mL, can have antiviral activity against NDV, but such concentrations were also linked to cytotoxicity, thus restricting their translational applicability (48). The postulated mechanisms include disruption of the nuclear transport mediated by importin α1/β, though observed mechanistically through cell models, and do not provide clinical antiviral efficacy (48).

Another important viral pathogen of poultry is the ILTV, and its prevalence has been reported to be around 13% in commercial flocks within Ethiopia (49). The information on the activity of ivermectin against ILTV is limited. There has been a limited observational report of clinical improvement in infected laying hens after the administration of ivermectin, but these studies are not reinforced by controlled studies on antiviral activity and should be treated as initial observations rather than evidence of antiviral activity (50).

IBV is still a significant problem in poultry production, with the prevalence rates estimated to be at about 40% in broiler chickens in Spain (51). Unlike NDV and ILTV, experimental studies have repeatedly shown that ivermectin lacks inhibitory effect against IBV *in vivo* models, meaning that this drug does not have any significant antiviral effect on this pathogen (52).

Additionally, the antiviral evidence in poultry is typified by a paucity of experimental data, extensive use of *in vitro* systems, and is not supported by a strong *in vivo* or field-based demonstration. As a result, the presence of antiviral effects in the literature should be seen as exploratory or mechanistic instead of actionable, and the use of ivermectin in the health of poultry has been based on antiparasitic use rather than antiviral intervention.

### 5.3 Viral diseases of equines and bovines

The viral pathogens in this subsection differ significantly in epidemiologic significance and in the quality of available antiviral evidence. In these studies, the disruptive effect of the importin α/β-mediated nuclear transport is a common mechanistic theme, although the relevance, in translation, has yet to be determined in any other system besides preclinical models.

#### 5.3.1 Importin α/β-mediated mechanistic evidence of viruses (preclinical *in vitro* and experimental models)

Hendra virus is a zoonotic pathogen that mostly affects horses and has an estimated incidence of about three cases annually in Australia, with a case fatality rate of about 100% according to national surveillance records (53). There is preclinical evidence that ivermectin has the potential to block importin a which is a host protein involved in viral replication, but the evidence is mechanistic and has not been supported by *in vivo* or clinical evidence (54).

Another category of mechanistic antiviral effects investigated is equine herpesviruses. The prevalence of equine herpesvirus (EHV) is extremely high, and it is estimated that EHV-1 can occur in up to 60% of horses across the world, and the prevalence of EHV-2 is estimated at about 66% (55, 56). The partial inhibition of viral replication has been observed in experimental research in the EHV-1 Jan-E strain but not in the Rac-H strain, showing strain-specific variation in response (57). Theorized mechanisms once again include interference with importin α/β-dependent nuclear transport, which adds to the overall mechanistic consistency that has been seen with a variety of viral systems (57).

#### 5.3.2 Bovine respiratory and enteric viruses: *in vitro* evidence with minimal translational context

There are a number of bovine viruses that have been studied mainly in cell-based systems. In 2022, the prevalence rate of Bovine Respiratory Syncytial Virus (BRSV) was reported at 10.8% in Polish cattle (58). *In vitro* experiments using MDBK cell lines have shown that BRSV replication is suppressed following ivermectin exposure, and that this inhibition can be attributed to importin α/β inhibition (59).

Equally, Bovine Parainfluenza Virus Type 3 (BPIV-3) has been reported with a prevalence of between 0.64 to 13.5% amongst European studies (58, 60). *In vitro* studies on the MDBK cells demonstrated that the replication of BPIV-3 was inhibited upon exposure to ivermectin (59).

In Poland, Bovine Coronavirus (BCoV) has been found in 32.4% of cattle (58). *In vitro* experiments in MDBK cells have shown that the BCoV replication is inhibited, and the mechanism of action is likely to be the interference of nuclear transport with the processes of importin α/β (59).

Bovine Viral Diarrhea Virus (BVDV) also has an inconsistent prevalence, with 0.8% in North America, Europe, and Australia, and more than 1.6% in West Asia (61). MDBK cell *in vitro* studies have demonstrated the inhibitory effects of ivermectin on the replication of the BVDV (59). In these respiratory and enteric bovine viruses, the antiviral effect is so far limited to cell culture models, without any field or clinical effect results.

#### 5.3.3 Exploratory experimental evidence of herpesviruses and related pathogens

Serological and region-specific surveys have shown that the bovine herpesvirus 1 (BoHV-1) is locally circulating, with a prevalence rate of about 5.4% in cattle (62). *In vitro* tests showed that viral replication in MDBK cells was inhibited after exposure to ivermectin (59).

In localized field and serological studies, 21.43% of cattle were found to be infected with varicellovirus bovinealpha 1 (BoAHV-1) (63). The study conducted *in vitro* with MDBK and BT cell lines reveals that ivermectin can suppress the development of the virus (64).

Alcelaphine herpesvirus 1 (AHV-1) causes Malignant Catarrhal Fever, which leads to high levels of herd mortality with rates of up to 10%/year in Kenya (65). *In vitro* experiments with BT1621 cells indicate that viral replication is inhibited by ivermectin and that the latter is mediated by mechanistic proof but has not been validated in a translational context (66).

#### 5.3.4 Viruses of limited or non-cell-based evidence

In Mexico, approximately 3.4% of the cattle have been reported to have Infectious Bovine Rhinotracheitis Virus (IBRV) (67). Experimental trials on a rabbit model showed that ivermectin inhibited viral replication, but did not affect viral binding and entry, which is suggestive of a post-entry mechanism of action (68).

#### 5.3.5 Critical synthesis

Antiviral effects of ivermectin have been reported in equine and bovine viral systems in a variety of preclinical and *in vitro* models, and host nuclear transport inhibition is often suggested to be the mechanistic underpinning. Nonetheless, the strength of evidence, depending on viruses, is still mostly experimental, lacks strain specificity, relevance of dosing, and lacks strong *in vivo* or field validation. It should therefore be understood that these results are mechanistic or hypothesis-generating as opposed to reflecting potentially clinically useful antiviral action in veterinary practice.

### 5.4 Emerging and miscellaneous viruses

Viral hemorrhagic septicemia virus (VHSV) is a highly pathogenic virus that affects many aquatic species, leading to significant economic losses in the fishing and aquaculture industries. Serological surveys of the region have also documented antibody-positive results in cattle with up to 72.4% prevalence rates that cast doubt on the history of exposure and potential inter-species transmission instead of active infection (65). *In vitro* experiments with Epithelioma Papulosum Cyprini (EPC) cells have also indicated that VHSV replication is inhibited following the exposure of the cells to ivermectin, although this is still in its infancy as an experimental observation (66). In addition, *in vivo* experiments in infected olive flounders have also indicated better survival following the use of ivermectin, but this may be seen as model-specific preliminary evidence rather than evidence of a validated therapeutic role (66).

Based on national and regional surveillance data, Usutu virus (USUV) has been reported in 105 confirmed cases in humans, and other region-specific outbreaks have been reported in birds and mosquitoes (69). *In vitro* studies have demonstrated a level of inhibition of USUV replication in Vero CCL-81, A549, and TME-R cells (70). The antiviral mechanism appears to involve the inhibition of flaviviral non-structural protein 3, which is crucial for viral replication (71).

Porcine parvoviruses (PV) have an estimated prevalence of 19.6 in both region-specific surveillance and cross-sectional studies, also demonstrating localized infection burden rather than worldwide prevalence. In cell culture, ivermectin interfered with parvovirus replication by blocking IMPα/β-dependent nuclear import of the B19V NS1 protein, resulting in reduced viral replication (72). The proposed mechanism involves disruption of nuclear localization, although further research is needed to confirm this effect (73).

Papillomavirus (PV) infections affect approximately 4.4% of cattle worldwide (74). In certain observational contexts, regression or remission of the lesions due to treatment with ivermectin has been reported in both bovine and caprine papillomatosis (74–77). Nevertheless, these results are rather heterogeneous, the underlying mechanism is still unclear, and existing data do not provide a proven antiviral indication (74–77).

Viruses mentioned in this section are added to indicate emerging, atypical, or multi-species disease situations, which are also pertinent to veterinary practice in a One Health approach. Although there are pathogens which afflict aquatic species and others that are zoonotic, their presence highlights the pathways of environmental exposure and cross-species interfaces that go beyond the conventional livestock infections. They have been included with this purpose, though, not to imply direct clinical relevance in all species, but to put ivermectin-related antiviral observations in the animal-environment-human interface into context.

Taken together, these results, also summarized in Table 2, cannot be accepted as clinical antiviral activity. The effects of observed inhibition are highly virus-specific, are often concentration-dependent, and are often at levels of exposure that are not attainable or safe during the normal course of veterinary dosage schedules. Conversely, experiments in animal models or in systems of physiological relevance have often shown minimal, non-reliable, or no antiviral activity, even of FMDV, IBV, and BTV (33, 52, 64). Such negative and null results are especially informative in clinical decision-making because they draw attention to the failure of *in vitro* antiviral inhibition to translate into significant *in vivo* results. Therefore, the existing evidence suggests that the antiviral activity of ivermectin is still experimental, virus-specific, and has pharmacological limitations, and should not be compared to its proven antiparasitic activity in animal clinical practice (32, 59). The most important translational obstacles are pharmacokinetic barriers, species-specificity of drug distribution, lack of *in vivo* validation, and absence of controlled clinical trials with meaningful antiviral effects in veterinary disease.

## 6 Pharmacology and mechanisms

The antiparasitic properties of ivermectin are primarily due to its binding to glutamate-gated chloride (GluCl) channels, which are found in nerve and muscle cells of invertebrate organisms and are absent in mammals. Upon binding, ivermectin causes a prolonged phase of chloride ion influx, resulting in hyperpolarization, neuronal firing inhibition, paralysis, and ultimately, death of the parasite (15). The selectivity accounts for the high therapeutic index of ivermectin in veterinary medicine. The drug can also increase the activity of the *γ*-aminobutyric acid (GABA)-gated chloride channel at higher concentrations, to further inhibit nervous system dysfunction in susceptible parasites (2). New developments in molecular parasitology have provided a wealth of new mechanistic insight into ivermectin beyond its traditional definition as a GluCl channel agonist. Single-channel recordings and structural experiments reveal that ivermectin selectively stabilizes the open conformations of high-affinity GluCl channels and hence increases the duration of chloride influx and results in long-term neuromuscular paralysis in parasitic nematodes (15, 78). These biomechanical observations are consistent with the electrophysiological data of the effect of ivermectin on hyperpolarization and long-term inhibitory signaling in *Haemonchus contortus* and other species.

Additionally, ivermectin can interact with other receptors at higher concentrations, including glycine, histamine, and nicotinic acetylcholine receptors, which further contribute to its antiparasitic effects (79, 80). The structural studies have offered high-resolution information regarding the GluCl-ivermectin interaction. Cryo-electron microscopy has shown that ivermectin binds to transmembrane domains of the channels and tends to stabilize its open shape, inducing prolonged influx of chloride into the cell (5). Along with its known action on glutamate-gated chloride channels, ivermectin has also been suggested to have secondary effects on membrane ion homeostasis (80). These hypothetical ionophoric or membrane-disruptive activities are a hypothesis-generating process that is not well described in comparison with GGCC-mediated neuromuscular paralysis.

At the parasite’s end, the development of transcriptomic and genomic studies has shown that human exposure to ivermectin leads to adaptive responses in transporter proteins, detoxification pathways, and neuronal gene expression. Comparative transcriptomics of resistant and susceptible isolates of *H. contortus* indicates the upregulation of ABC transporters and altered expression of GluCl subunits (5, 81). Recent studies involving parasitology also demonstrate how the P-glycoproteins regulate the safety, pharmacokinetic, and resistance formation of ivermectin in the helminth and arthropods (4, 5). Combining these mechanistic observations, ivermectin can be viewed as a multitarget molecule because it can act by functioning via direct interaction with channels, as well as indirectly changing host-pathogen signaling systems.

While the anti-parasitic mechanisms of ivermectin are well-studied, the antiviral activity of ivermectin is complex and virus-specific. The major mechanism is via inhibition of nuclear transport using the importin a/b system, which is probably used for the transport of viral proteins into the host cell nucleus, a mechanism on which most viruses are dependent. With its inhibiting action on this pathway, ivermectin disrupts replication of bovine respiratory syncytial virus (BRSV), bovine parainfluenza virus (BPIV-3), and porcine circovirus 2 (PCV2) (59). In porcine reproductive and respiratory syndrome virus (PRRSV), ivermectin seems to work at an earlier stage of the infection cycle and prevents viral entry into alveolar macrophages. Controlled trials in pigs have found decreased viremia, a decrease in clinical signs, and increased survival after ivermectin treatment during PRRSV infection (6). For DNA viruses like pseudorabies virus (PRV), ivermectin inhibits viral DNA synthetic polymerases directly, thereby stopping the genome replication and viral propagation (28). Furthermore, research on capripox viruses, such as sheep pox virus (SPPV) and lumpy skin disease virus (LSDV), indicates that ivermectin works at several phases of the viral life cycle, from binding to entrance to replication, underscoring the broad spectrum of its antiviral mechanisms (40).

There is also emerging evidence that ivermectin has an impact on host-side biological pathways in treated animals. The use of ivermectin has been reported to have a largely untapped immunomodulatory effect through oxidative-stress modulation, cytokine-modulation, and cellular redox signal changes in viral papillomatosis and inflammatory disease models (74, 75). Such interactions between hosts and pathways can affect other clinical outcomes besides the antiparasitic effect, especially when it concerns viral infection, immune dysregulation, or complicated host–parasite interactions. Combining these molecular discoveries with species-specific pharmacokinetic profiles can be used to provide opportunities for optimizing the dosage and resistance-reducing approaches in veterinary medicine.

## 7 Adverse events and drug interactions

Ivermectin is valuable for the control of veterinary diseases, but there are potential downsides, including ecological impacts (82), development of drug resistance (15, 83, 84), and potential side effects, including drug interactions.

Certain species have a higher frequency of genetic mutations, which can increase ivermectin toxicity. Approximately 1/3 of border dogs are homozygous for a mutated form of the MDR1 gene, which makes them susceptible to IVM toxicity (85, 86). This risk may be attenuated by titrating the dose for at-risk species or possibly by genetic testing for MDR1 alleles prior to IVM prescription (87).

In general, ivermectin has a large therapeutic margin in the majority of food-producing species when administered at the recommended dosage (85–87). Conversely, a smaller safety margin has been reported in some dog species, young animals, and species with a limited production of P-glycoprotein-mediated drug efflux, with neurotoxicity developing at relatively low levels of exposure.

The off-label use of ivermectin is also occasionally applied in poultry species and wild animals. In such species, there is a lack of pharmacokinetic and safety information. The variability in the distribution, metabolism, and excretion of drugs can lead to the risk of toxicity. These doubts raise concerns regarding neurological adverse events, residual presence, and unwanted ecological exposure (82, 88).

Applying a pharmacovigilance perspective, the reporting of adverse events related to the use of ivermectin is imperative (15, 83, 84). It is especially significant when the drug is used off-label, has drug interactions, or is used in genetically prone animals. Enhancing the post-marketing surveillance would enhance the early detection of rare or context-dependent toxicities. It is also through such reporting that evidence-based changes in dosing recommendations and regulatory advice can be informed.

Drug interactions with IVM are a concern for some animals being treated with other medications. Ivermectin is usually well tolerated in veterinary doses approved for use, but there are circumstances when the drug–drug interactions can be clinically significant. The potential interactions are most pertinent in cases when ivermectin is co-administered with compounds which affect the systemic exposure and central nervous system penetration by P-glycoprotein transports or by the cytochrome P450-mediated metabolism (2, 88). Species/breed-specific vulnerability also changes the risk of interaction, most significantly in dogs with gene mutations in MDR1, in which defective drug efflux is more likely to result in neurotoxicity when interacting agents are present (85–87). Based on this, cautious use of concomitant drugs and genetic risk factors should be considered whenever ivermectin is administered outside the routine antiparasitism prophylaxis. Table 3 presents the summary of the reported ivermectin drug-interaction patterns in the context of veterinary pharmacology, with reference to the mentioned literature for contextual synthesis (88–111).

**Table 3:** IVM’s interactions with other drugs.

Drug	Effect when given with Ivermectin
Verapamil	Increased IVM Cmax and AUC with topical application (89)
Loperamide	Increased IVM plasma levels and AUC after subcutaneous administration; higher availability in the liver and small intestine (90)
Itraconazole	Enhanced IVM bioavailability after administration of itraconazole (91)
Ketoconazole	Higher plasma levels of IVM (92)
Cyclosporin A	Increased neurotoxicity; higher brain concentrations of IVM (93)
Trifluoperazine	Increased neurotoxicity (93)
Fexofenadine	Ivermectin pretreatment lowers AUC when fexofenadine is given orally (94)
Cetirizine	Ivermectin pretreatment 12 h before cetirizine increased AUC, Cmax, MRT, and t1/2 (95)
Doramectin	AUC of doramectin was significantly higher when administered alone vs. with moxidectin or ivermectin (96)
Moxidectin	Co-administration with ivermectin produces a higher AUC for moxidectin (not always significant) (96)
Albendazole	Increased AUC of albendazole sulfoxide (active metabolite). Ivermectin plasma AUC was 88% higher after coadministration compared to treatment with ivermectin alone (97)
Triclabendazole	Ivermectin plasma availability was 3-fold higher, and elimination was significantly delayed (98)
Rafoxanide	Increased absorption and delayed elimination of ivermectin (99)
Pyrantel Pamoate	No significant drug interaction found (100)
Doxycycline	No known issues with co-administration (101)
Diethylcarbamazine	No known issues with co-administration (102)
Levamisole	Not recommended; can cause convulsions, vomiting, dyspnea, and death (103)
Fenbendazole	No known issues with co-administration (104)
Abamectin	Potential for side resistance development (88) Combination affects milk pharmacokinetics (reduced half-life and residence time) for clorsulon (105)
Closantel	No known issues with co-administration (106)
Clorsulon	Reduced half-life and residence time of clorsulon in milk when treated with IVM (105)
Praziquantel	No known issues with co-administration (107)
Spinosad	Increased risk of ivermectin toxicity (108)
Lidocaine	Ivermectin antagonized the appearance of lidocaine-induced convulsions (109)
Strychnine	Ivermectin antagonized the appearance of strychnine-induced convulsions (109)
Pentylenetetrazol	Ivermectin protected rats from the convulsant effects of pentylenetetrazol (110)
Diazepam	Ivermectin exhibited anticonvulsant effects in mouse seizure models, acting through GABAergic and KATP channel mechanisms, and its efficacy was enhanced in the presence of diazepam (111)

## 8 Pharmacokinetics and dosing

Ivermectin has a high level of species-specific pharmacokinetic variability, which is determined by the lipophilicity, formulation, route of delivery, and interaction with transporters and co-comitant drug absorption. Subcutaneous and pour-on administration have been implicated in the species that produce food, including cattle, where changing peak plasma concentrations and extended tissue retention, a feature of absorption caused by formulation and long partitioning of lipid into adipose tissue, is evident (96). These properties contribute to sustained exposure but also add interindividual variability that makes it difficult to predict dosing in the field.

Systemic exposure is further modulated by the route of administration across species. Injectable formulations tend to produce more predictable absorption curves, though oral has been shown to be affected by gastrointestinal physiology and, in ruminants, rumen dynamics. There is further variation of dermal absorption and environmental factors with the topical (pour-on) formulation. The contribution of efflux transporters to these variations is emphasized by experimental data: inhibition of P-glycoprotein has a significant impact on the absorption of the drug dose to the skin and systemic exposure in rat models, which reveals transporter-mediated compensation of ivermectin bioavailability (89).

Drug–drug interactions and genetic determinants also have an effect on pharmacokinetic behavior. Oral ivermectin disposition is affected in horses by concomitant administration with antihistamines, including fexofenadine and cetirizine, which is in line with the well-known transporter-mediated interactions with systemic exposure (94, 95). When administered concomitantly with itraconazole in sheep, it changes the disposition and systemic handling in the gastrointestinal tract, based on metabolic and transporter pathway inhibition (91). In dogs with MDR1 mutations, which have defects in transporter operations, the central nervous system becomes more penetrated and has a greater risk of neurotoxicity after an oral dose (87). Moreover, in calves and sheep, when used together with other highly lipophilic anthelmintics, e.g., rafoxanide, the co-administration causes a redistribution of tissues and changes in pharmacokinetic behavior due to competitive lipid partitioning and protein-binding effects (99).

These pharmacokinetic properties in animals that produce food have a direct connection in terms of residue persistence and withdrawal. Due to the lipophilicity of ivermectin and the long tissue retention, the drug may be found as residues in edible tissues and milk after it has been administered, especially when using long-acting injectable or topical preparations. The time of withdrawal is thus species, formulation, and administration route dependent and plays a vital role in dosing decisions to guarantee food safety and regulatory compliance (4, 81). Kinetic residue depletion should be put into consideration when introducing ivermectin into the parasite-controlling programs in livestock systems (81). Table 4 provides a summary of key determinants of ivermectin pharmacokinetic variability between species and administration conditions and highlights the reasons why dosing choices should be species- and formulation-specific.

**Table 4:** Ivermectin efficacy, pharmacokinetics, and comparative potency across veterinary species.

Category	Species/parasite/virus	Dose/route	Reported findings/PK value / comparative result	References
Efficacy	*Haemonchus contortus* (ruminants)	SC/Pour-on	Great efficacy, 80–99% in controlled studies; resistance is being experienced in certain areas.	(5, 148)
	Bovine hypodermosis	Long-acting SC	Almost 100 percent effective; timing is seasonal	(17, 19)
	Mange in rabbits	SC	Fast rates of recovery and cure.	(9)
	Sarcoptic mange in pigs	SC	Increased effect in combination with quercetin.	(21)
	Babesia/Theileria spp.	SC / Oral	*In vitro* and *in vivo* inhibition.	(22)
	Fur mites (rodents)	Oral	Colonial eradication was successful.	(24, 25)
	Ticks/ectoparasites	SC	High fatality during field study.	(151)
	Biting midges (Culicoides)	SC/Pour-on	Decrease in blood-feed and survival.	(145, 146)
Pharmacokinetics	Cattle	SC/Pour-on	Unstable Cmax and long-term tissue persistence.	(96)
	Horses	Oral	PK changes in the presence of antihistamines (fexofenadine, cetirizine)	(94, 95)
	Sheep	SC	Gastrointestinal disposition changes in response to interaction with itraconazole.	(91)
	Rats	Pour-on	Raised absorption with verapamil.	(89)
	Dogs	Oral	MDR1 mutation affects CNS exposure and toxicity.	(87)
	Calves & sheep	SC	PK difference in co-administration with rafoxanide.	(99)
Comparative potency vs. other macrocyclic lactones	Ivermectin vs. moxidectin in GI nematodes	SC/Pour-on	Moxidectin is better in resistant strains.	(117)
	Ivermectin vs. doramectin vs. moxidectin (Merino sheep)	SC	Moxidectin and Doramectin are slightly greater in AUC, and persistence is longer.	(96)
	Ivermectin vs. thiabendazole (*in vitro*)	Mixed	The entomopathogenic nematodes exhibit variation in patterns of susceptibility.	(149)
	Ivermectin vs. closantel (cattle)	SC	Formulation combination enhances protection against nematodes and flukes.	(106)
	Ivermectin vs. Albendazole	SC	Combination therapy changes PK and could increase efficacy.	(97)

## 9 Resistance and ecological aspects

### 9.1 Epidemiology and emergence of ivermectin resistance

One of the major issues that has come to light in veterinary parasitology is ivermectin resistance, which has become especially problematic in intensively farmed livestock systems where parasites have to endure prolonged selective pressure due to repeated and prophylactic use of ivermectin (112–115). The development of anti-drug resistance is troubling, but manageable through the use of alternative treatments (116, 117) or drug combinations (118, 119). The most extensively recorded cases of resistance include gastrointestinal nematodes of cattle, sheep, and goats, in which diminishing efficacy has been linked with treatment failure and greater production losses (113–115). These tendencies indicate that mechanistic knowledge is required to guide sustainable control of parasites.

### 9.2 Production-system and geographic diversity in resistance

Notably, there is a great geographical variation in the development and severity of ivermectin resistance, which is attributable to the regional disparities in the types of parasites, climatic conditions, production systems, and the practice of drug use. The highest concentrations of resistance are reported to have been repeatedly observed in small ruminant systems in Australia, South America, and portions of Southern Africa, where macrocyclic lactones have been used intensively and frequently, and where their use has offered a very strong selective pressure (113–115). These geographical differences suggest that ivermectin resistance is not a universal phenomenon across the world, and it should be examined in the context of local epidemiology and control.

### 9.3 Cellular and molecular mechanisms of resistance

**Figure 1:**
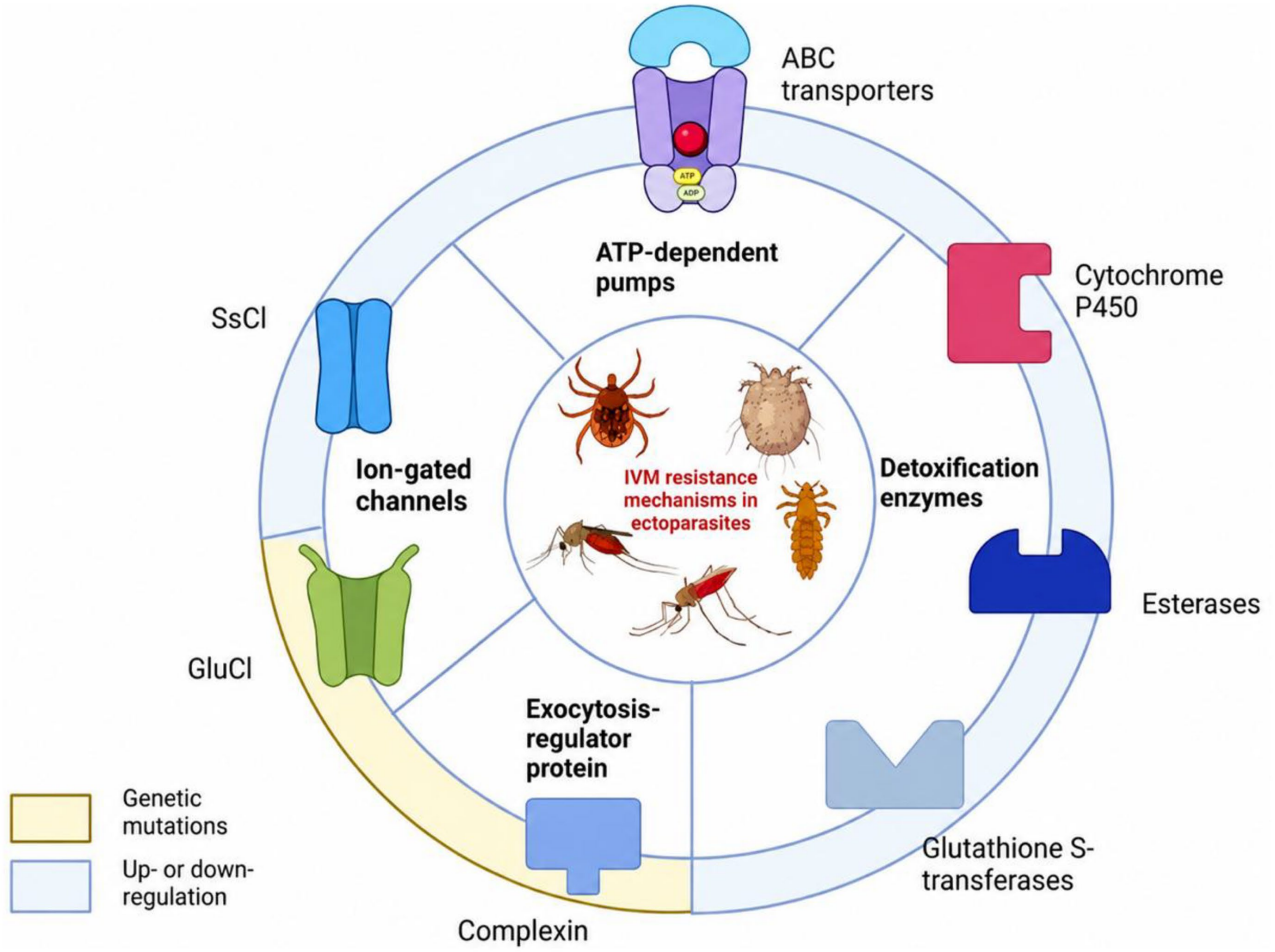
Mechanisms of IVM drug resistance in ectoparasites. Reproduced from (120) under a Creative Commons 4.0 license (http://creativecommons.org/licenses/by/4.0/).

The molecular mechanism of resistance to ivermectin is multifactorial. The changes in glutamate-gated chloride channel subunits, the enhanced expression of efflux transporters, and the changes in parasite metabolism have been identified as the probable contributors to decreased susceptibility to drugs in experimental and field studies (15, 120–126). Studies of resistant isolates by transcriptomics and genomic methods have repeatedly shown the upregulation of ATP-binding cassette (ABC) transporters and P-glycoproteins, and in so doing have been supportive of their role in the limitation of intracellular drug accumulation (122, 126). These results are valuable information about the biology of resistance, but also highlight the complexity of the evolution of resistance. Drug combinations can be selected to find the direction of steepest descent in the fitness landscape for the parasite. For the case of IVM’s mechanism of action of glutamate-mediated endotoxicity, resistance mechanisms can emerge through altered conformation of the glutamate receptor, or other mechanisms (Figure 1) (120).

### 9.4 Resistance detection and surveillance

Ivermectin resistance detection in the field is the least developed in the case of helminths, where phenotypic assays, especially the fecal egg count reduction test (FECRT), remain the most frequently used practical method of detecting diminished drug susceptibility in livestock systems (15). Due to the potential limitations in sensitivity of phenotypic techniques, interpretation limits, and early detection, there has been a growing focus on complementary molecular techniques, such as the measurement of glutamate-gated chloride channel changes and modified expression of ATP-binding cassette transporter- and P-glycoprotein-related pathways related to reduced susceptibility (120, 122, 124, 126). Interpretation of reduced response to treatment can be complicated by regional differences in parasite populations, level of use of drugs and production practices, and context-specific surveillance becomes particularly relevant (15, 113–115). Uniform reporting of phenotypic response, molecular markers, host species, and treatment conditions would enhance comparability between studies and aid the early identification of new trends of resistance (120, 122, 124, 126). Nevertheless, the concept of resistance surveillance cannot be considered as applicable only to gastrointestinal nematodes. Ectoparasites are increasingly also considered within the wider ivermectin-resistance context, even though surveillance in these systems is not as standardized as in helminth surveillance (120). The increased surveillance view is in line with the extended resistance biology of ivermectin against helminths and arthropods and promotes combined surveillance strategies in veterinary practice (4, 5, 15, 120–126).

### 9.5 Strategies

Some experimental methods have been investigated on how to overcome the resistance of ivermectin, such as applying ABC-transporter inhibitors and metabolic enzyme modulators (126–128). Although this kind of intervention has been shown to cause increased sensitivity of ivermectin to laboratory systems and in *ex vivo* systems, they are still proof-of-concept approaches. The current clinical use of ABC transporter inhibitors is not feasible because of the toxicity, absence of species-specific data on safety, and regulatory limitations. In turn, these methods are to be understood as the methods of explaining the resistance mechanisms, but not as the possible therapeutic resources in veterinary practice.

### 9.6 Environmental/ecological consequences of using Ivermectin

Simultaneously with the development of resistance, the ecological effects of using ivermectin have been reported in a variety of terrestrial and aquatic ecosystems. The remaining ivermectin released in feces continues to be released into the environment and has neurotoxic effects on non-target invertebrates, such as dung beetles, earthworms, and springtails (129–133). These organisms are very important in nutrient cycling, soil aeration, and organic matter decomposition, and their decay has been associated with distorted ecosystem functions (132–134). It has been shown that field studies have shown a lower rate of dung degradation and alteration in invertebrate community structure after exposure to ivermectin (135–138). The selective treatment has, therefore, been suggested to reduce the ecological impact, but maintain antiparasitic effectiveness through mitigation strategies such as specific selective treatment, timing in administration, and pasture management strategies (139–141).

### 9.7 Stewardship and sustainable control of antiparasitics

The contextualization of ivermectin resistance in the context of the antiparasitic stewardship framework connects the resistance management, diagnostic surveillance, dose control, and sustainability. Evidence-based stewardship focuses on the correct dosage, non-prophylactic use, resistance-based treatment approaches, and selective interventions oriented towards maintaining efficacy, with minimal ecological impact (116–119, 139–141). In this context, resistance is not merely viewed as a pharmacological constraint, but rather as a consequence of ecological and management stress, which in turn supports the argument that needs to be integrated approaches to parasite control that are effective, ecologically, and evolutionarily sustainable.

## 10 One Health perspective (conceptual framework)

One Health perspective is the conceptual framework present in this review that incorporates the use of ivermectin, development of resistance, exposure to the environment, and sustainability in the long term. Instead of conceiving these domains as independent outcomes, the One Health framework puts the pharmacological efficacy, resistance selection, and ecological impact into a context of interdependent consequences of veterinary drug use in animal-environment-human systems. The conceptual model gives a coherent way of understanding the different mechanistic, clinical, and ecological results of the review. It helps to critically assess the use of ivermectin in veterinary medicine, not only on the basis of the outcomes of individual animals, but also based on the sustainability of livestock and human health, environmental integrity, and sustainability. Although the ecological impacts of ivermectin residues were explained in detail above, its application in a One Health context is seen in the interaction of these effects with regulatory measures, stewardship, and system resilience in the long term (135–139).

From a regulatory perspective, ivermectin defines the issues of balancing treatment need with unintended effects on the environment. The persistence of residues in dung and soil has raised an increasing level of concern among the regulatory authorities, especially in the areas of withdrawal times, off-label use, and environmental risk assessment (137–140). It has been demonstrated in field and experimental studies that ivermectin metabolites may appear several weeks and in some instances several months following administration in dung (137–140). This can take place at a time when the residue levels will be high enough to cause neurotoxicity in vulnerable non-target invertebrates. Risk-based frameworks in a number of jurisdictions are currently focusing on selective treatment targeting, seasonal administration limits, and monitoring of the effect of non-target species in veterinary medicinal products authorization and post-marketing surveillance (139–141).

Stewardship approaches that are determined by policy are thus at the heart of ensuring that the ecological effects are reduced without compromising antiparasitism effectiveness. These comprise incorporation of parasite control into herd-health planning, less dependence on prophylactic mass treatment, and conformity of the dosing practice with the epidemiological risk instead of convenience (139–141). Selective treatment methods and a timely approach to administration are considered the most immediate and effective interventions among the existing mitigation options (139–141). On the contrary, larger-scale pasture management and biodiversity observation strategies are critical, but they usually demand more infrastructural back-ups and regulatory capital to execute successfully. These strategies show a move towards a drug-centered control of managing systems, which is in line with One Health.

Notably, the development of resistance and impact on the environment are not distinct issues but are connected by common factors, such as excessive use, standard approach to dosing, and lack of ecological feedback in the decision-making process. One Health-oriented governance stimulates unified activity in the field of veterinary practice, agricultural policy, and environmental protection, so that the antiparasitic use is effective and sustainable (136–141). Enhancing regulatory guidelines, surveillance infrastructure, and multidisciplinary collaboration will be necessary for the sustained administration of ivermectin and related chemicals in integrated animal health systems.

The presence of these issues is further exacerbated by climate change, pasture intensification, and continuous degradation of biodiversity that changes the dynamics of parasites and routes of drug exposure (135–141). These collectively make non-target species more susceptible and highlight the fact that ivermectin stewardship should be part of the One Health and environmental sustainability model.

## 11 Limitations and controversies

There are a number of limitations associated with this review which need to be taken into account during the interpretation of the evidence at hand. This is because much of the mechanism and antiviral literature around ivermectin in the veterinary setting is based on *in vitro* systems or experimental animals, and therefore cannot be directly extrapolated to clinical applications (36–40, 59), which adds to the existence of heterogeneity in published results. Various study designs, dosing schedules, outcome variables, and species-specific pharmacokinetics also limit cross-study comparability (2, 4).

The second constraint is associated with resistance and ecological data that do not have an equal distribution across parasite species, host animals, and geographic locations. Despite the growing molecular-level understanding of its resistance mechanisms, such as changes in glutamate-gated chloride channels and increased efflux transporter activity, the process of translating such results into practical, field-scale resistance control has not been accomplished yet (15, 120–126, 142). Equally, ecological impact research usually targets sentinel taxa or short time-endpoints, and long-term and ecosystem-wide outcomes are less characterized (129–138). In aquatic and semi-aquatic environments, the existing data are predominantly species and safety-oriented and restrict the extent to which generalization by taxa is possible (143).

There is also controversy on how to interpret non-antiparasitic effects, especially the findings on antiviral and immunomodulatory effects. They are often concentration-dependent and context-specific, which leads to the question of their biological relevance at accepted veterinary doses (36–40). On this basis, these effects can be most appropriately considered as hypothesis-generating and not evident of known therapeutic activity.

## 12 Discussion

This review gives a synthesis of the use of ivermectin in veterinary medicine beyond the descriptive compilation that directly connects the molecular mechanisms and clinical use, evolution of resistance, ecological impact, and stewardship concerns (2, 4, 15, 112–141). Instead of addressing these areas separately, the review shows how dosing, formulation, and frequency of use decisions affect the outcomes in both the biological and environmental levels.

The uses of ivermectin may be classified based on the degree of evidence. Its antiparasitic activity is a clinically proven activity with several decades of supporting mechanistic and field data (2, 4, 142). Conversely, immunomodulatory or adjunctive impacts can be described as new fields of research that have a scanty translational validation (36–40). The antiviral effect is still very speculative in nature, with the majority of supporting evidence being obtained on *in vitro* systems at exposure levels that cannot be obtained under conventional veterinary dosing regimens (36–40, 59).

Regulatory and policy-wise, these differences are essential towards the direction of approval, stewardship policies, and off-label use. Modern regulatory frameworks are markedly shifting the focus towards risk-based review, pharmacovigilance, and environmental effects in addition to the therapeutic efficacy to assess veterinary medicinal products (129–135). In line with this, evidence-proportional use of ivermectin is necessary to balance the clinical benefit, resistance management, and environmental protection goals (136–141).

Although this review uses a qualitative narrative synthesis, there were some quantitative data available, which were compared to find the ranges, contrasts, and trends between species, regions, and production systems. Claims of antiparasitic activity in gastrointestinal nematodes against ivermectin in ruminants have been reported to be high in experimental field studies, but to be significantly decreased in regions where it has developed resistance, especially on small ruminants in South America, Africa, and Australia, where resistance levels have been reported to be over 50% in some cases (5, 15, 81). Conversely, its effectiveness in managing ectoparasitoids like mange mites, hypodermosis larvae, and its capacity to impact previously inaccessible organisms like phytoplankton has similarly shown high efficacy (>90) when used correctly in the field (16, 19, 21). The quantitative prevalence data also prove the presence of significant regional heterogeneity in parasite burden, as the prevalence of gastrointestinal nematodes and the related economic losses differ across a wide range depending on climate, intensity of management, and methodology of parasite surveillance (11, 13, 18). Limited *in vitro* tests of antiviral drugs have demonstrated high *in vitro* antiviral activity, which can typically result in more than 90% reduction in viral replication. However, it is a virus-specific effect and usually only occurs at doses incompatible with normal veterinary administration, which is supported by *in vivo* and field data suggesting little or no clinical effect (36–40, 52, 64). This review integrates quantitative ranges and trends into a qualitative framework to distinguish mechanistic or experimental signals from clinically and ecologically relevant veterinary evidence, thereby increasing interpretation clarity without suggesting inappropriate equivalence among study types.

One of the key contributions of this review is that it provides a clear difference between the proven antiparasitic activity of ivermectin and experimental or hypothesis-generating data. Although the molecular mechanism of interaction of ivermectin with glutamate-gated chloride channels is thoroughly understood (142), extrapolation to applications outside the antiparasitic setting, such as antiviral or immunomodulatory applications, should be approached with care due to variations in exposure levels, biology of the targets, and host-pathogen interactions (36–40, 59). With this differentiation, the review demarcates clear boundaries in evidence that are usually overlapped in the literature.

Resistance biology and practical implications in management are also incorporated in the review. Molecular concepts of channel changes and efflux transporter upregulation offer valuable perspectives on resistance emergence but also explain why pharmacological reversal interventions are mostly experimental (15, 120–128). Sustainable management of parasites thus relies more on steward-based management rather than increasing drug combinations or drug intensity.

Lastly, ecological and environmental factors are integrated as part of veterinary pharmacology and not as peripheral issues. This review contextualizes the use of ivermectin within the context of One Health through placing residue persistence, non-target toxicity, and resistance selection within the greater contexts of stewardship and regulatory concerns that require long-term sustainability and therapeutic efficacy (129–141).

Together, such an integrative method enables the review to act as a conceptual framework, rather than a catalogue of studies, highlighting where evidence is strong, where uncertainty still exists, and where future research and policy focus are needed.

## 13 Future research directions

Therefore, in order to support scientifically supported veterinary usage, subsequent studies should focus on thorough environmental monitoring, comparative head-to-head trials with alternative macrocyclic lactones, resistance-predicting biomarker establishment, and testing of field-ready stewardship technologies. These combined will help enable a more balanced approach toward the integration of therapeutic efficacy, resistance management, and environmental protection within a One Health approach.

Simultaneously, future studies must focus on the combination of pharmacokinetic-pharmacodynamics models and field epidemiology to optimize the dose between species and production systems. The combination of resistance surveillance, treatment outcomes, and environmental residue tracking, which would be performed through longitudinal studies, would enable the earlier identification of developing resistance and unintended ecological impacts. More standardization of study designs, outcome measures, and reporting frameworks is also required to enhance cross-study comparability and to facilitate evidence-based decision making. These strategies together would enable the translation of mechanistic and experimental understanding into a practical and scalable approach, which would maintain the effectiveness of the ivermectin but would make the veterinary practice in line with the long-term sustainability objectives.

## 14 Conclusions and perspectives

Ivermectin has proven to be an indispensable tool in veterinary medicine, offering broad-spectrum efficacy against a variety of parasitic infections in numerous animal species. Its primary mechanism, involving the binding to glutamate-gated chloride channels in invertebrates, ensures its effectiveness and safety across different applications. The drug’s versatility extends beyond antiparasitic uses, showing potential antiviral activities, although these effects require further elucidation and validation. Despite its widespread use, concerns remain regarding the development of resistance, particularly in parasites, and the potential toxicological effects in border collies and other dogs.

The main strength of the current evidence base is the uniformity of antiparasitic activity of ivermectin in different species and production systems. Nonetheless, there remain significant limitations, such as the preclinical or *in vitro* nature of antiviral data, a large degree of interspecies pharmacokinetic disparity, and controlled clinical trials to support non-parasitic indications. These are the limitations that restrict direct clinical translation and point towards those areas where evidence is still early and not conclusive.

The One Health lens perspective on ivermectin as long-term value needs to be determined by weighing the effectiveness of parasite control against the reduction of resistance and environmental sustainability (129–133). There is thus the necessity to integrate veterinary practice, regulatory control, and ecological sustainability so as to assure that ivermectin is not only clinically effective but also environmentally responsible in the integrated animal-health systems (134–141).

Future research needs should then be directed towards the generation of effective *in vivo* and clinical data on new applications, the enhancement of resistance monitoring systems, and the elimination of enlightenment in stewardship, wherein therapeutic benefit is considered alongside ecological and public health considerations. These gaps will need to be addressed as critical to the continued usefulness of ivermectin, and to prevent overextension beyond its currently validated evidence base.
